# Real‐world management of adjuvant systemic melanoma therapy: Multi‐center survey of 51 DeCOG skin cancer centers

**DOI:** 10.1111/ddg.15963

**Published:** 2025-11-22

**Authors:** Markus Reitmajer, Elisabeth Livingstone, Lucie Heinzerling, Kai‐Martin Thoms, Frank Meiss, Markus V. Heppt, Anja Gesierich, Konstantin Drexler, Friedegund Meier, Max Schlaak, Andrea Forschner, Lisa Zimmer

**Affiliations:** ^1^ Department of Dermatology University Hospital Tuebingen Tuebingen Germany; ^2^ Department of Dermatology University Hospital Essen Essen Germany; ^3^ Department of Dermatology and Allergology University Hospital LMU Munich Munich Germany; ^4^ University Medical Center Goettingen Goettingen Germany; ^5^ Department of Dermatology Medical Center – University of Freiburg Faculty of Medicine University of Freiburg Freiburg Germany; ^6^ Department of Dermatology Uniklinikum Erlangen Friedrich‐Alexander‐University Erlangen‐Nürnberg (FAU) Erlangen Germany; ^7^ Comprehensive Cancer Center Erlangen‐European Metropolitan Area of Nuremberg (CCC ER‐EMN) Erlangen Germany; ^8^ Bavarian Cancer Research Center (BZKF) Uniklinikum Erlangen Friedrich‐Alexander‐University Erlangen‐Nürnberg (FAU) Erlangen Germany; ^9^ Department of Dermatology University Hospital Wuerzburg Wuerzburg Germany; ^10^ Department of Dermatology University Hospital Regensburg Regensburg Germany; ^11^ Department of Dermatology Faculty of Medicine and University Hospital Carl Gustav Carus Technische Universität Dresden Dresden Germany; ^12^ Skin Cancer Center at the University Cancer Centre Dresden and National Center for Tumor Diseases Dresden Germany; ^13^ Department of Dermatology Venereology and Allergology Charité‐Universitätsmedizin Berlin corporate member of Freie Universität Berlin and Humboldt‐Universität zu Berlin Berlin Germany

**Keywords:** Adjuvant therapy, follow‐up care, immune checkpoint inhibitor (ICI), PET/CT, recurrence, targeted therapy (TT)

## Abstract

**Background:**

Adjuvant immune checkpoint inhibitors (ICI) and targeted therapy (TT) have revolutionized treatment management for patients with melanoma. However, the current German S3 guideline does not differentiate between patients with and without adjuvant therapy in its recommendations for imaging intervals and follow‐up monitoring, leading to variability in clinical practice. This study provides an overview of surveillance practices among skin cancer centers in the *German Dermatologic Cooperative Oncology Group* (DeCOG).

**Methods:**

A survey was sent to 80 skin cancer centers in Germany, Austria and Switzerland on November 22, 2023. Responses received by March 10, 2024 were analyzed descriptively.

**Results:**

Fifty‐one responses (64%) were received. Forty centers (78%) reported deviations from the guideline. In stage IIB, 34 centers (67%) conduct imaging including CT scans prior to and after adjuvant therapy. Post‐adjuvant therapy, 36 centers (71%) conduct imaging according to the guideline. Over 90% of the centers offer adjuvant ICI therapy if progression occurs on adjuvant TT and vice versa. In stage IV, after relapse while on adjuvant ICI, 32 centers (63%) would offer adjuvant TT.

**Conclusions:**

Surveillance practices differed among participating centers and often do not align with the current guideline. A consensus based consistent approach would benefit both patients and physicians.

## INTRODUCTION

The approval of immune checkpoint inhibitors (ICI) and targeted therapies (TT) for melanoma has led to periodic revisions of the evidence‐based German S3 guideline titled “Diagnosis, Therapy, and Follow‐up of Melanoma” (Registry Number: 032/024OL) with the last update in July 2020.^1^ In 2020, the recommendation concerning the adjuvant setting of the tumor stages III–IV (AJCC 2017) with anti‐PD1 antibodies and BRAF/MEK‐inhibitors as TT has been implemented.^1^, ^2^, ^3^, ^4^ The results of the KEYNOTE‐716 study and the CheckMate 76K study have led to the approval of pembrolizumab (in 2022) and nivolumab (in 2023) for the adjuvant treatment of stage IIB and IIC melanoma and both are now widely used in stage IIB/C.^1^, ^5^, ^6^ These recommendations have not yet been integrated into the current S3 guidelines but are already offered to patients as adjuvant therapy in clinical practice. Besides the stage‐dependent follow‐up recommendation of the German S3 guideline, there are no specific recommendations for follow‐up before, during, and after the completion of adjuvant systemic therapy. For example, according to current guideline recommendations, no radiological diagnostics are performed in stage IIB. Particularly with regard to the early detection of distant recurrence during adjuvant therapy, the question arises whether follow‐up recommendations, including radiological imaging and laboratory diagnostics, should be adjusted independently of tumor stage – similar to the approach taken in the respective adjuvant approval studies.^1^ Increasing the frequency of imaging and extending it to stage IIB are being considered, as these are expensive treatments with significant side effects. It is essential to ensure that the patient genuinely benefits from them. Early detection of metastases could be crucial, and treatments that prove ineffective yet potentially toxic should be promptly discontinued. On the other hand, increased radiation exposure, including non‐specific findings and the psychological impact on patients must also be considered.^7^, ^8^, ^9^, ^10^

Furthermore, positron emission tomography/computed tomography (PET‐CT) has emerged as a reliable imaging tool for primary diagnostic and follow‐up, demonstrating superiority compared to conventional computed tomography (CT) in terms of sensitivity.^11^, ^12^, ^13^, ^14^, ^15^ However, its nationwide availability and cost coverage vary among the centers, not only within the DeCOG.^13^ Current German S3 guideline recommendations include radiographic imaging in stage IIC–IV every 6 months during the first 3 years. In stage IIB, only ultrasound diagnostics are recommended, with no CT or MRI scans. There is no distinction made regarding whether the patient is currently undergoing adjuvant therapy, has previously received adjuvant therapy, or has never had adjuvant therapy.^1^

In July 2023, the *Survivorship Committee* of the *German Dermatologic Cooperative Oncology Group* (DeCOG) was established to foster open and intensive communication with patient representatives.^16^ This dialogue revealed that patients are often confused and concerned due to the varying imaging intervals and monitoring procedures implemented across different skin cancer centers during adjuvant therapy and following relapse. Therefore, the Committee decided to invite all skin cancer centers within the DeCOG to participate in a survey to assess the approach of each center, with the aim of providing a comprehensive summary report thereafter.

### Aims

This study aimed to assess and evaluate the real‐world management of patients with melanoma and adjuvant systemic therapy within the DeCOG skin tumor centers in Germany, Austria and Switzerland through a survey, examining the extent to which the centers deviate from the stage‐dependent follow‐up recommendations of the S3 melanoma guideline and whether they implement intensified diagnostics before, during, and after adjuvant systemic therapy.

## METHODS

### Study design

Led by the Survivorship Committee of the DeCOG, a questionnaire was developed within the committee (online supplementary Table S1). The questionnaire regarding the management of adjuvant therapy was divided into two sections. The first part focused on diagnostic management during and after adjuvant therapy, while the second part addressed decision‐making in case of disease recurrence. On November 22, 2023, the questionnaire was distributed to the medical directors of all 80 certified skin cancer centers within the DeCOG network, covering Germany (n = 72), Austria (n = 4), and Switzerland (n = 4). Two reminders were sent on December 11, 2023, and January 17, 2024. All responses received by March 10, 2024, were included in the analysis.

### Statistical analysis

The questionnaire was created in Microsoft^®^ Excel® 2016 MSO (Version 2403). Descriptive analysis was conducted with IBM^®^ SPSS^®^ Statistics (version 28.0.0.0). Incomplete questionnaires were included using the available responses. Graphs were generated using GraphPad Prism^®^ (version 10.0.1).

## RESULTS

### Response‐rate

Fifty‐one responses from 80 certified skin cancer centers in Germany, Austria and Switzerland, were received (response rate of 63.8%) (Table 1). Forty‐nine responses were sent from Germany, and one response each from Switzerland and Austria. More than half of the responding certified skin cancer centers (n = 31, 60.8%) were located at university hospitals.

**TABLE 1:** Summary of survey results from 51 skin cancer centers regarding imaging during adjuvant therapy.

	n	%
**Response‐rate**		
Contacted certified skin cancer centers within the DeCOG	80	100
Skin cancer centers that have responded (= cohort)	51/80	63.8
Not answered	29/80	36.3
**Imaging during adjuvant therapy according to melanoma stages in guideline recommendations**		
Yes	11/51	21.6
Individual intervals	40/51	78.4
**Imaging after adjuvant therapy according to guideline recommendations**		
Yes	36/51	70.6
Individual intervals	15/51	29.4

### Most centers practice more intensive monitoring and use more frequent intervals during adjuvant therapy than recommended in the current guideline

**FIGURE 1:**
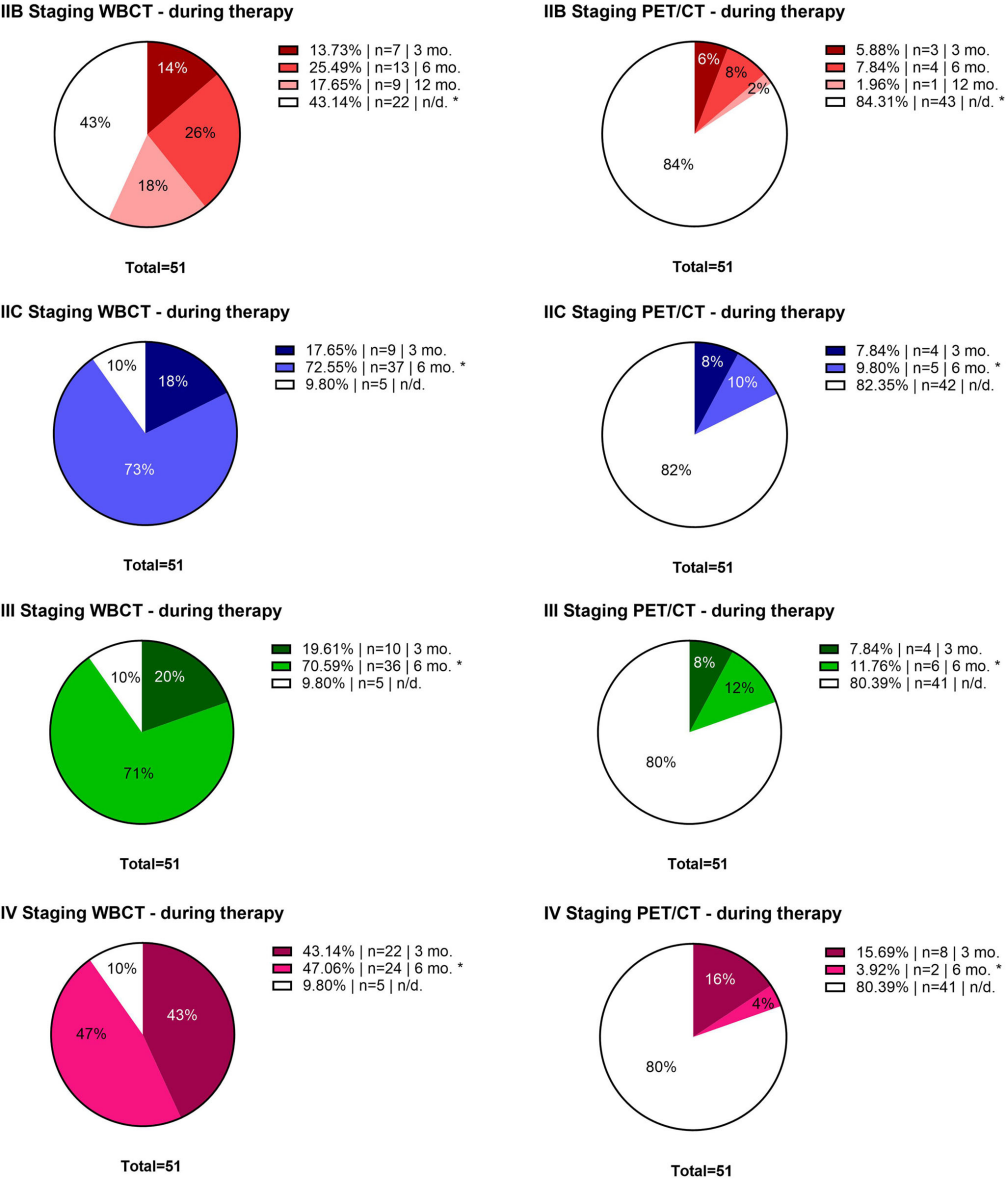
Follow‐up examinations and intervals during adjuvant ICI. Preferred imaging modality during adjuvant ICI. The recommended interval in the current guideline is marked with an asterisk (*). *Abbr*.: WBCT, whole‐body computed tomography; WB PET/CT, whole‐body positron emission tomography/computed tomography; n/d, not done

Regarding the imaging intervals during adjuvant therapy, eleven centers (22%) adhere exactly to the imaging intervals outlined in the current guideline (Table 1). This means that in stage IIB, imaging with CT or magnetic resonance imaging (MRI) are not routinely performed, and in stages IIC‐IV, whole‐body CT (WBCT), cerebral magnetic resonance imaging (cMRI)/cerebral computed tomography (cCT) are conducted at 6‐month intervals. The other 40 skin cancer centers (78%) deviated from the guideline with closer imaging intervals in stage IIB‐IV (Figure 1, Table 2).

**TABLE 2:** Detailed intervals for imaging during and after adjuvant therapy.

Imaging before the start of adjuvant therapy								
WBCT/PET‐CT	Stage IIB		Stage IIC		Stage III		Stage IV	
	*n*	*%*	*n*	*%*	*n*	*%*	*n*	*%*
Yes, WBCT before the start of adjuvant therapy	24	47.1	41	80.4	39	76.5	39	76.5
Yes, PET‐CT before the start of adjuvant therapy	3	5.9	4	7.8	5	9.8	5	9.8
Yes, imaging with PET‐CT or WBCT (depending on the case) before the start of adjuvant therapy	7	13.7	5	9.8	6	11.8	6	11.8
No imaging before the start of adjuvant therapy	17	33.3	–	–	–	–	–	–
Not answered	–	–	1	2.0	1	2.0	1	2.0

Imaging during adjuvant therapy								
WBCT	Stage IIB		Stage IIC		Stage III		Stage IV	
	*n*	*%*	*n*	*%*	*n*	*%*	*n*	*%*
3‐monthly interval	7	13.7	9	17.6	10	19.6	22	43.1
6‐monthly interval	13	25.5	37	72.5	36	70.6	24	47.1
12‐monthly interval	9	17.6	–	–	–	–	–	–
Not done	22	43.1	5	9.8	5	9.8	5	9.8
Not answered	–	–	–	–	–	–	–	–

Imaging during adjuvant therapy								
WB PET/CT	Stage IIB		Stage IIC		Stage III		Stage IV	
	*n*	*%*	*n*	*%*	*n*	*%*	*n*	*%*
3‐monthly interval	3	5.9	4	7.8	4	7.8	8	15.7
6‐monthly interval	4	7.8	5	9.8	6	11.8	2	3.9
12‐monthly interval	1	2.0	–	–	–	–	–	–
Not done	43	84.3	42	82.4	41	80.4	41	80.4
Not answered	–	–	–	–	–	–	–	–

Imaging after adjuvant therapy								
WBCT	Stage IIB		Stage IIC		Stage III		Stage IV	
	*n*	*%*	*n*	*%*	*n*	*%*	*n*	*%*
3‐monthly interval	2	3.9	3	5.9	4	7.8	10	19.6
6‐monthly interval	7	13.7	47	92.2	47	92.2	41	80.4
12‐monthly interval	–	–	–	–	–	–	–	–
Not done	42	82.4	1	2.0	–	–	–	–
Not answered	–	–	–	–	–	–	–	–

*Abbr*.: WBCT, whole‐body computed tomography; PET‐CT, positron emission tomography/computed tomography; WB PET/CT, whole‐body positron emission tomography/computed tomography; n, number of centers

All centers (n = 50) that answered this section of the questionnaire perform imaging in stages IIC, III, and IV before the start of adjuvant treatment. In stage IIB, 34 centers (67%) perform a complete imaging, which includes whole body imaging and cerebral imaging at least at the beginning and after the completion of adjuvant therapy. Imaging intervals during adjuvant therapy vary between centers. Twenty centers (40%) additionally use WBCT for imaging during adjuvant therapy. In stages IIC and III, about 20% (9 centers in stage IIC and 10 centers in stage III) conduct imaging every 3 months instead of the guideline‐recommended 6‐month intervals for stage IIC–IV patients. The number of centers using a shortened 3‐month interval for monitoring increases in stage IV with no evidence of disease (NED). Here, 22 centers (43%) perform imaging every 3 months. PET‐CT imaging is used by eight centers (16%) during adjuvant therapy in stage IIB, by nine centers (18%) in stage IIC and ten centers (20%) in stages III–IV with (Table 2).

### Imaging after discontinuation of adjuvant therapy mostly corresponds to the guideline

**FIGURE 2:**
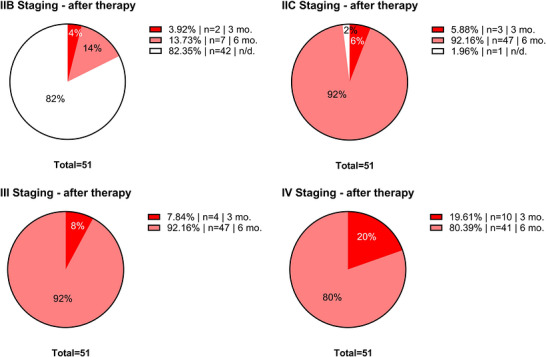
Follow‐up examinations and intervals after adjuvant ICI.

Regarding the imaging modalities and intervals after adjuvant therapy, 36 centers (71%) adhere to the imaging recommendations. The current guideline makes no distinction in its recommendation based on whether or not adjuvant therapy has been given. The other 15 skin cancer centers (29%) mainly shortened the intervals (Table 2, Figure 2). In stage IIB, nine centers (18%) continue radiological imaging at 3‐ or 6‐month intervals. Concerning stages IIC and III, 47 centers (92%) adhere to the 6‐month intervals. In stage IV, 41 centers (80%) adhere to the 6‐month intervals, 10 centers (20%) have closer intervals of every 3 months (Table 2, Figure 2).

### Decision‐making in in the event of recurrence during adjuvant therapy

**FIGURE 3:**
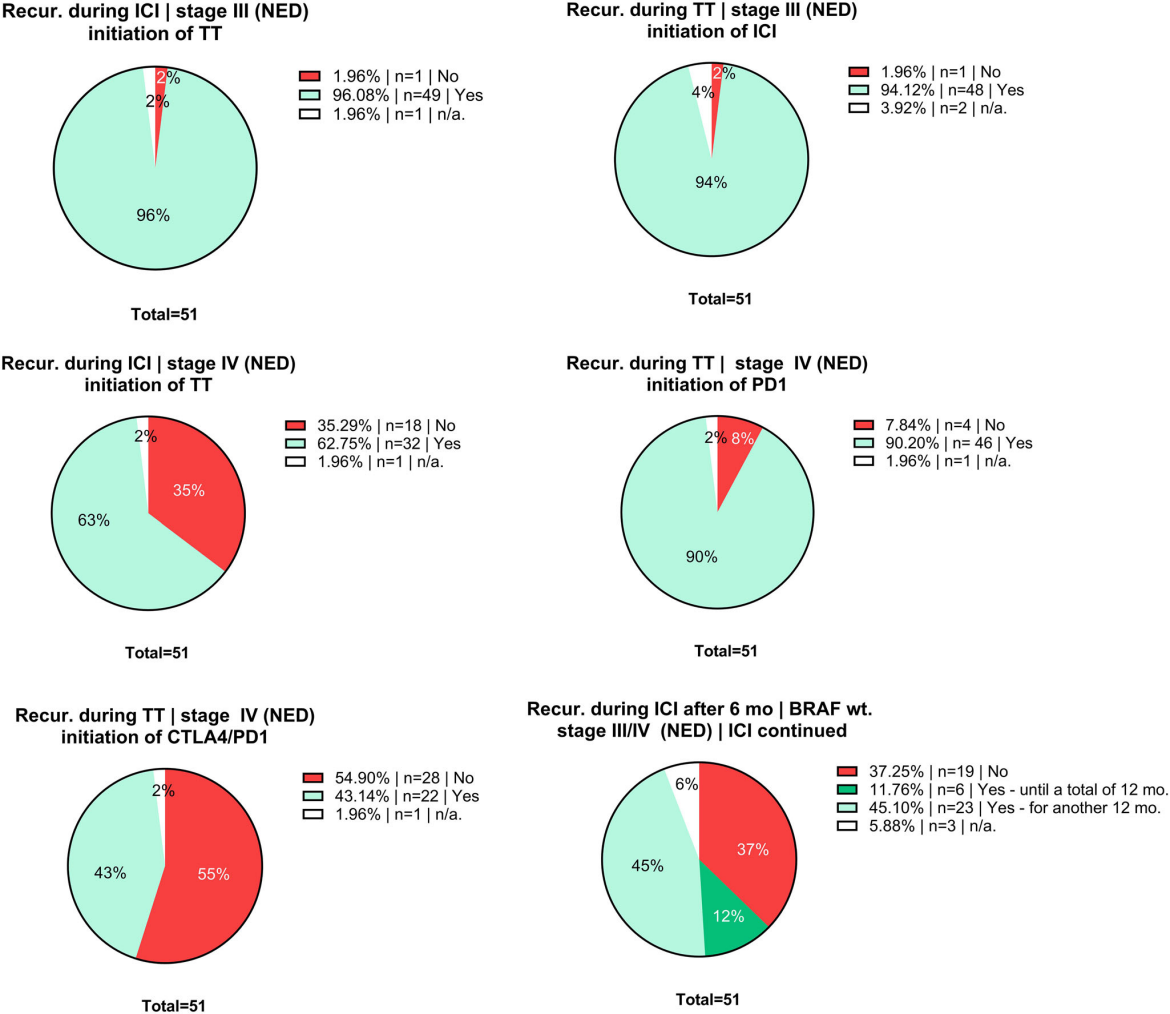
Decision‐making in the event of recurrence during adjuvant therapy. *Abbr*.: Recur., recurrence; MD, medical doctor; n/a, no data available; NED, no evidence of disease; ICI, immune checkpoint inhibitor

In the second section of the questionnaire, we presented typical scenarios occurring in real‐world adjuvant therapy management. All of these scenarios involve decision‐making in the event of disease recurrence during adjuvant therapy (Table 3, Figure 3).

**TABLE 3:** Decision‐making in the event of recurrence during adjuvant therapy.

	n	%
**Recurrence during ICI | stage III, NED | initiation of TT**		
No	1/51	2.0
Yes	49/51	96.1
Not answered	1/51	2.0
**Recurrence during TT | stage III, NED | initiation of ICI**		
No	1/51	2.0
Yes	48/51	94.1
Not answered	2/51	4.0
**Recurrence during ICI | stage IV, NED | initiation of TT**		
No	18/51	35.3
Yes	32/51	62.7
Not answered	1/51	2.0
**Recurrence during TT | stage IV, NED | initiation of PD1**		
No	4/51	7.8
Yes	46/51	90.2
Not answered	1/51	2.0
**Recurrence during TT | stage IV, NED | initiation of CTLA4/PD1**		
No	28/51	54.9
Yes	22/51	43.1
Not answered	1/51	2.0
**Recurrence during ICI after 6 mo. | stage III/IV, NED | *BRAF*‐wt. | ICI continued**		
No continuation	19/51	37.3
Yes, until a total of 12 mo.	6/51	11.8
Yes, for another 12 mo.	23/51	45.1
Not answered	3/51	5.9

*Abbr*.: ICI, immune checkpoint inhibitor; TT, targeted therapy; NED, no evidence of disease; PD1, programmed cell death protein 1 inhibitor; CTLA4, cytotoxic T‐lymphocyte‐associated protein 4 inhibitor; BRAF‐wt, BRAF wild type; mo., months; n, number of centers

Forty‐eight centers (94%) offer adjuvant ICI therapy after complete excision of a recurrence during adjuvant TT in stage III melanoma. Conversely, 49 centers (96%) reported that they would offer adjuvant TT after complete resection of a recurrence that was detected during ICI treatment in *BRAF*‐mutated stage III patients (Table 3, Figure 3).

In case of relapse with resectable distant metastases in a stage IV (NED) patient undergoing ICI treatment, 32 centers (63%) would offer adjuvant TT after successful R0 resection (off‐label in stage IV, NED). In a similar situation involving the development of resectable metastases during adjuvant TT, 46 centers (90%) would offer PD‐1 (programmed cell death protein 1) monotherapy after resection, while 22 centers (43%) would also discuss the option of a combined ICI therapy with ipilimumab/nivolumab (off‐label).

In case of resectable metastasis in *BRAF* wild‐type patients after ≥ 6 months of adjuvant ICI, 19 centers (37%) would not continue adjuvant ICI treatment after R0 resection. Six centers (12%) would continue ICI for an additional 6 months after resection, and 23 centers (45%) would extend adjuvant ICI for an additional 12 months after R0 resection (Figure 3).

## DISCUSSION

The era of ICI and BRAF/MEK inhibitors has rapidly and extensively transformed the management of melanoma treatment. With their introduction into the adjuvant setting, and their application now extending to stage IIB‐–IIC, new questions and demands arise.^2^, ^3^, ^4^, ^5^ Based on current evidence, adjuvant treatment is associated with improved relapse‐free survival and distant‐metastasis‐free survival, but a significant improvement in overall survival (OS) has not been demonstrated to date, with the exception of the potential OS benefit of dabrafenib/trametinib in patients with *BRAF* V600E mutant melanoma.^2^, ^3^, ^17^, ^18^ The number needed to treat (NNT) to prevent one relapse in stage IIB/IIC melanoma is approximately 7.81.^19^, ^20^ Currently, international guidelines vary on recommendations for adjuvant treatment and follow‐up care in advanced‐stage melanoma.^1^, ^21^, ^22^, ^23^, ^24^ The ASCO Guideline, the ESMO Clinical Practice Guidelines, and the NCCN (National Comprehensive Cancer Network) Guideline have been updated and now recommend adjuvant pembrolizumab or nivolumab for patients with resected stage IIB or IIC melanoma. However, the current German S3 guideline “Diagnosis, Therapy, and Follow‐up of Melanoma”, which was last updated in 2020, has not yet addressed this topic, as the approval of adjuvant therapy for stage IIB–IIC occurred in 2022/2023 (online supplementary Table S2). The 2025 ESMO Clinical Practice Guideline says that there is no consensus on the optimal follow‐up schedule or the utility of imaging with resected melanoma. It proposes that respective national guidelines should be consulted and adjusted as required, considering available resources, particularly after 3 years of follow‐up. In contrast to the German S3 guideline, the ESMO guideline already recommends staging including ultrasound, CT and/or PET scans, and cMRI for patients with stage IIB melanoma to ensure proper tumor assessment.^1^, ^25^ All guidelines lack specific recommendations regarding imaging intervals during and after adjuvant therapy, resulting in different procedures among skin cancer centers.^1^, ^23^, ^25^, ^26^, ^27^

In this multi‐center survey analyzing survey results from 51 skin cancer centers from three countries, nearly 80% of the skin cancer centers deviate from the current stage‐adapted guideline‐recommendations during adjuvant therapy. Twenty‐nine of the 51 centers conduct intensive imaging procedures during adjuvant therapy in stage IIB with up to 3 monthly intervals. To our knowledge, no data is available addressing the relevance of imaging before, during, and after adjuvant therapy in stage IIB melanoma in real‐world. Initial imaging and at least one follow‐up imaging after the completion of therapy are essential for establishing a baseline (including potential pathological changes) prior to treatment initiation. This approach enables a more accurate assessment of the effectiveness and potential toxicities of adjuvant ICI therapy, facilitating better evaluation and ensuring comprehensive follow‐up care. For instance, a patient may have pre‐existing lung structural changes before the commencement of adjuvant immunotherapy. Without a baseline, it remains unclear whether these changes were pre‐existing or induced by the immunotherapy. In the case of pre‐existing lung structural alterations, a risk‐benefit evaluation of adjuvant therapy could have been discussed with the patient. The phase III studies leading to the approval adjuvant ICI, required imaging every 3–6 months until up to year 5 after randomization.^2^, ^3^, ^5^ The CheckMate‐76K trial and the KEYNOTE‐716 trial demonstrated improved recurrence‐free survival (RFS) in the group treated with the PD‐1 inhibitor compared to placebo.^5^, ^6^ However, a relapse rate of 20.3% after 3 years was observed in the stage IIB pembrolizumab group and 22% in the stage IIB/C nivolumab group, despite treatment with ICI.^20^, ^28^ Considering the entire cohort of stage IIB patients, including those who have not received therapy and therefore have not had the potential opportunity for improved RFS, it seems reasonable to conduct radiological imaging not only on patients undergoing adjuvant ICI therapy but also on those without it. Patients without adjuvant ICI remain at a higher risk of recurrence, making imaging beneficial for early detection in this group as well. In the non‐adjuvant setting, between 40%–60% of patients are primary non‐responders to ICI. If we hypothetically assume that patients in the adjuvant setting respond similarly to those in the non‐adjuvant setting, approximately 50% of patients undergoing adjuvant ICI may not benefit from the ICI treatment.^29^, ^30^, ^31^ Additionally, regular imaging may facilitate the early detection of side effects, such as ICI‐related pneumonitis, potentially helping to prevent further organ damage.^32^, ^33^ Since late onset immune‐related adverse events (irAE) are frequent in ICI therapy, treatment despite progression poses a risk of unnecessary toxicity.^34^, ^35^ Thus, frequent imaging can improve the benefit‐risk ratio.

After discontinuation of adjuvant ICI in stage IIB, nine centers continue radiographic imaging even in the post‐adjuvant period. However, when discussing post‐adjuvant follow‐up imaging in stage IIB, it is essential to acknowledge potential consequences of an increased radiation exposure, including non‐specific findings and the psychological impact on patients when awaiting the scan results.^7^ Increased radiation exposure during cancer follow‐up care, particularly in young women, is a significant concern and is associated with increased breast cancer risks later in life.^8^, ^9^, ^10^ Contrary to the current German Guideline, the imaging intervals in stage IV (NED) are more frequent than 6‐monthly in many centers. Over 40% of the centers conduct imaging with WBCT 3‐monthly during adjuvant therapy, and even after the completion of adjuvant therapy approximately one‐fifth of the centers continues imaging in 3‐monthly intervals.

In the second part of the questionnaire, we evaluated procedures of real‐world scenarios in the context of adjuvant therapy decision‐making. In the scenario of a *BRAF*‐mutated patient with locoregional but resectable relapse (stage III) and adjuvant ICI or TT, more than 90% of centers would switch after complete removal of relapse to the other adjuvant therapy (ICI → TT / TT → ICI). In contrast, the answers concerning subsequent “second‐line adjuvant” therapy in a stage IV, NED patient, differed markedly. Approximately 60% of the centers would recommend off‐label adjuvant TT after removal of relapse, if patients had adjuvant ICI therapy before. However, the decision of the other centers, not to offer TT can be attributed to the lack of approval for TT in the adjuvant setting for stage IV melanoma (NED) and insufficient data regarding adjuvant TT in stage IV patients. The current German S3 guideline recommends for patients with tumor stage IV (NED) adjuvant ICI only.^1^, ^36^ Retrospective data suggest a benefit in terms of recurrence‐free survival at the expense of toxicity for “second‐line” adjuvant TT after failure of adjuvant ICI in stage III patients.^37^, ^38^ A retrospective multicenter analysis from the skin cancer registry ADOreg showed a benefit of combined adjuvant ICI therapy (anti‐PD1/anti‐CTLA4 ICI) in case of relapse upon adjuvant TT.^37^ The IMMUNED study revealed that both anti‐PD1 monotherapy and combined ICI significantly improved recurrence‐free survival of stage IV melanoma patients with NED compared to placebo. Overall survival, however, was only significantly better for patients receiving combined ICI.^39^ Despite these data, about 90% of centers offered “only” PD‐1 monotherapy after resection, and less than half of the centers (43%) offer adjuvant combined ICI therapy possibly due to approval limited to unresectable disease.

Overall, this multicenter study provides important insights into the real‐world management of patients with melanoma and adjuvant systemic therapy. Management varies between the different centers and discrepancies exist between the recommendations of the current guideline and the real‐world. Given the high cost of adjuvant ICI, the potentially detectable side effects in imaging scans and the chance of avoiding potential irreversible damage due to irAE, as well as the assessment of the benefit from the adjuvant therapy, it can be discussed to conduct imaging at least baseline, 3 or 6 monthly during and at the end of adjuvant ICI also in stage IIB patients.^32^, ^33^ The question of whether performing imaging procedures in the follow‐up program for high‐risk cutaneous melanoma improves overall survival through earlier detection is currently being investigated in the prospective randomized multicenter TRIM study (Trial to Assess the Role of Imaging During Follow‐up After Radical Surgery of Stage IIB‐C and III Cutaneous Melanoma, NCT 03116412).^40^ A preliminary interim analysis indicates no benefit from imaging in the follow‐up program for high‐risk patients.^41^ However, so far, only a few patients have completed the five‐year follow‐up period, with study completion estimated for December 31, 2028.^40^, ^41^ Early detection of disease recurrence might enable earlier initiation of subsequent therapies at probably lower tumor load and LDH levels, which could improve outcomes and survival.^42^, ^43^, ^44^

We observed relevant differences between the participating centers in their surveillance schemes, highlighting the need to determine the best approach. With ongoing rapid developments and increasing treatment options for advanced melanoma, there is a need for continuous revisions of the German S3 guideline. A consensus based consistent approach among skin cancer centers would be beneficial for both patients and physicians.

## FUNDING

MR received funding as part of the Junior Clinician Scientists Program of the University of Tübingen (application no. 523‐0‐0). Otherwise, this research has not received any specific grants from public, commercial or non‐profit organizations.

## CONFLICT OF INTEREST STATEMENT

M.R. received travel support from Almirall Hermal and Pierre Fabre and funding as part of the Junior Clinician Scientists Program of the University of Tuebingen (application no. 523‐0‐0). L.H. received board honoraria from BMS, Immunocore, Novartis, and Therakos and supervises clinical studies within the institution (Agenus, BMS, Regeneron, Replimune, Huyabio International, Immunocore, IO Biotech, MSD, Pfizer, Pierre Fabre, Sol‐Gel Technologies), outside the submitted work. E.L. served as consultant and/or received honoraria from Bristol Myers Squibb, Merck Sharp & Dohme, Novartis, Pierre Fabre, Sanofi, Sunpharma, and Takeda and travel support from Bristol Myers Squibb, Pierre Fabre, Sunpharma, and Novartis, outside the submitted work. K.M.T. received speaker and advisory board honoraria from Bristol Myers Squibb, Merck Sharp & Dohme, Pierre Fabre, Novartis, Roche, Immunocore, Sanofi, Sun Pharma, Amgen, LEO, Galderma, Almirall, Candela, and Lilly, outside the submitted work. F.M. served as consultant and/or received honoraria from Novartis, Bristol Myers Squibb, Merck Sharp & Dohme, Pierre Fabre, Sanofi Genzyme, and Sun Pharma and travel support from Novartis, Sun Pharma, Pierre Fabre, and Merck Sharp & Dohme, outside the submitted work. M.V.H. received honoraria from MSD, BMS, Roche, Novartis, Sun Pharma, Sanofi, Almirall, Biofrontera, Infectopharm, Immunocore, and Galderma, outside the submitted work. A.G. served as consultant and/or received honoraria or travel costs from Almirall, Amgen, Bristol Myers Squibb, Immunocore, Merck Sharp & Dohme, Novartis, Pierre Fabre Pharmaceuticals, Pfizer, Roche, and Sanofi Genzyme, outside the submitted work. K.D. reports advisory roles for or received honoraria from Pierre Fabre Pharmaceuticals, Novartis, and BMS, outside the submitted work. F.M. received travel support and/or speaker's fees and/or advisory honoraria from Novartis, Roche, BMS, MSD, Pierre Fabre, Sanofi, and Immunocore and research funding from Novartis and Roche, outside the submitted work. M.S. reports advisory roles and travel support from BMS, MSD, Novartis, Pierre Fabre, Sun Pharma, Immunocore, and Kyowa Kirin, outside the submitted work. A.F. served as consultant to Novartis, MSD, BMS, Pierre Fabre, and Immunocore; received travel support from Novartis, BMS, and Pierre Fabre; received speaker fees from Novartis, BMS, Delcath, and MSD; and reports institutional research grants from BMS Stiftung Immunonkologie, outside the submitted work. L.Z. served as consultant and received honoraria from Bristol Myers Squibb, Merck Sharp & Dohme, Novartis, Pierre Fabre, Sanofi, and Sunpharma and travel support from MSD, BMS, Pierre Fabre, Sanofi, Sunpharma, and Novartis, outside the submitted work.

## Supporting information



Supplementary information
