# Supplementary material for: Real‐world management of adjuvant systemic melanoma therapy: Multi‐center survey of 51 DeCOG skin cancer centers
Source: J Dtsch Dermatol Ges. 2025 Nov 22;24(7):916–26. doi: 10.1111/ddg.15963 (PMC13340988; doi:10.1111/ddg.15963)
Supplement: Supplementary file 1 — Supplementary information [file DDG-24-916-s001.docx]

**SUPPLEMENTARY TABLE S1** Questionnaire.

| **How do you conduct your imaging examinations for adjuvant therapy?** | | | | | |
| --- | --- | --- | --- | --- | --- |
|  | | **Stage IIB** | **Stage IIC** | **Stage III** | **Stage IV** |
| **Imaging for adjuvant therapy** | During adjuvant treatment according to the guideline (Stage IIB without CT/MRT, IIC-IV every 6 months WBCT, cMRI or cCT) |  |  |  |  |
|  | During adjuvant treatment, different imaging intervals (then subsequent from line 57 to 66) |  |  |  |  |
| **CT thorax, CT abdomen, cMRI** | 1x before the start of therapy |  |  |  |  |
|  | Every 3 months during therapy |  |  |  |  |
|  | Every 6 months during therapy |  |  |  |  |
|  | 1x after completion of therapy |  |  |  |  |
| **PET-CT with cCT or cMRI** | 1x before the start of therapy |  |  |  |  |
|  | Every 3 months during therapy |  |  |  |  |
|  | Every 6 months during therapy |  |  |  |  |
|  | 1x after completion of therapy |  |  |  |  |
| **Imaging after completion of adjuvant therapy** | After completion of adjuvant therapy according to the guideline (Stage IIB without CT/MRI, IIC-IV every 6 months WBCT, cMRI or cCT for the first 3 years)) |  |  |  |  |
|  | Different imaging intervals: 0 = no imaging, 1 = imaging every 3 months for 3 years, free text |  |  |  |  |

| **If a *BRAF*-mutated patient under adjuvant immunotherapy or BRAF/MEK inhibitor therapy demonstrates locoregional progression but resectable progression, do you offer them the respective other adjuvant therapy after resection (and subsequent tumor-free Stage III)?** | | |
| --- | --- | --- |
|  | **Yes** | **No** |
| In the event of progression during ICI treatment, following surgery, BRAF/MEK therapy is offered if the patient remains in Stage III NED. |  |  |
| In the event of progression during BRAF/MEK-inhibitors, following surgery, ICI is offered if the patient remains in stage III NED. |  |  |
|  | | |
| **If a *BRAF*-mutated patient demonstrates resectable progression with distant metastases under adjuvant immunotherapy or BRAF/MEK inhibitor therapy, do you offer them the respective other adjuvant therapy after resection (and subsequent tumor-free Stage IV)?** | | |
|  | **Yes** | **No** |
| In the event of progression during ICI treatment, post-surgery, BRAF/MEK therapy is provided if the patient maintains Stage IV NED. |  |  |
| In the event of progression during BRAF/MEK therapy, post-surgery, ICI (PD1 Mono), is provided if the patient maintains Stage IV NED. |  |  |
| In the event of progression during BRAF/MEK therapy, post-surgery, ICI (PD1 +CTLA4), is provided if the patient maintains Stage IV NED. |  |  |
|  | | |
| **If a *BRAF* wild-type patient demonstrates resectable progression under adjuvant immunotherapy after receiving adjuvant immunotherapy for 6 months, do you offer them the continuation of adjuvant immunotherapy after resection (and subsequent tumor-free Stages III and IV)?** | | |
|  | **Yes** | **No** |
| Yes, a new course of immunotherapy for 1 year, counted from the time of resection. |  |  |
| Yes, continuation of immunotherapy for a total of 1 year, meaning for an additional 6 months. |  |  |
| No, discontinuation of immunotherapy and follow-up according to guidelines. |  |  |

**SUPPLEMENTARY TABLE S2** Comparison of the guidelines.

| Guideline | Recommendation of adjuvant PD-1 therapy in stage IIB/C included | Recommendation of adjuvant PD-1 therapy in stage III/IV included | Imaging recommendations during adjuvant therapy | Staging adapted imaging recommendations |
| --- | --- | --- | --- | --- |
| NCCN Guideline Melanoma: Cutaneous  Swetter et al. 2024 | Yes | Yes | No | Yes |
| Systemic Therapy for Melanoma: ASCO Guideline  Seth et al. 2023 | Yes | Yes | No | No |
| German S3 guideline “Diagnosis, Therapy, and Follow-up of Melanoma”  AWMF [last update 2020] | No | Yes | No | Yes |
| ESMO Clinical Practice Guidelines  Amaral et al. 2025 | Yes | Yes | No | Yes |

*The approval of adjuvant therapy occurred after the publication of the guideline.
